# Body mass index and risk of COVID-19 diagnosis, hospitalisation, and death: a cohort study of 2 524 926 Catalans

**DOI:** 10.1210/clinem/dgab546

**Published:** 2021-07-23

**Authors:** Martina Recalde, Andrea Pistillo, Sergio Fernandez-Bertolin, Elena Roel, Maria Aragon, Heinz Freisling, Daniel Prieto-Alhambra, Edward Burn, Talita Duarte-Salles

**Affiliations:** 1 Fundació Institut Universitari per a la recerca a l’Atenció Primària de Salut Jordi Gol i Gurina (IDIAPJGol), Barcelona, Spain; 2 Universitat Autònoma de Barcelona, Spain; 3 International Agency for Research on Cancer (IARC-WHO), 150 Cours Albert Thomas, 69008 Lyon, France; 4 Centre for Statistics in Medicine, NDORMS, University of Oxford; 5 Department of Medical Informatics, Erasmus University Medical Center, Rotterdam, Netherlands

**Keywords:** obesity, adiposity, SARS-CoV-2, hospitalisation, fatality, electronic health records

## Abstract

**Context:**

A comprehensive understanding of the association between body mass index (BMI) and COVID-19 is still lacking.

**Objective:**

To investigate associations between BMI and risk of COVID-19 diagnosis, hospitalisation with COVID-19, and death after a COVID-19 diagnosis or hospitalisation (subsequent death), accounting for potential effect modification by age and sex.

**Design:**

Population-based cohort study.

**Setting:**

Primary care records covering >80% of the Catalan population, linked to region-wide testing, hospital, and mortality records from March to May 2020.

**Participants:**

Adults (≥18 years) with at least one measurement of weight and height.

**Main outcome measures:**

Hazard ratios (HR) for each outcome.

**Results:**

We included 2 524 926 participants. After 67 days of follow-up, 57 443 individuals were diagnosed with COVID-19, 10 862 were hospitalised with COVID-19, and 2467 had a subsequent death. BMI was positively associated with being diagnosed and hospitalised with COVID-19. Compared to a BMI of 22kg/m ^2^, the HR (95%CI) of a BMI of 31kg/m ^2^ was 1.22 (1.19-1.24) for diagnosis, and 1.88 (1.75-2.03) and 2.01 (1.86-2.18) for hospitalisation without and with a prior outpatient diagnosis, respectively. The association between BMI and subsequent death was J-shaped, with a modestly higher risk of death among individuals with BMIs ≤19kg/m ^2^ and a more pronounced increasing risk for BMIs ≥40kg/m ^2^. The increase in risk for COVID-19 outcomes was particularly pronounced among younger patients.

**Conclusions:**

There is a monotonic association between BMI and COVID-19 diagnosis and hospitalisation risks, but a J-shaped one with mortality. More research is needed to unravel the mechanisms underlying these relationships.

This project is funded by the Health Department from the Generalitat de Catalunya with a grant for research projects on SARS-CoV-2 and COVID-19 disease organized by the Direcció General de Recerca i Innovació en Salut. This project has received support from the European Health Data and Evidence Network (EHDEN) project. EHDEN received funding from the Innovative Medicines Initiative 2 Joint Undertaking (JU) under grant agreement No 806968. The JU receives support from the European Union's Horizon 2020 research and innovation programme and EFPIA. The University of Oxford received a grant related to this work from the Bill & Melinda Gates Foundation

## Introduction

The coronavirus disease 2019 (COVID-19), the illness caused by the Severe Acute Respiratory Syndrome Coronavirus 2 (SARS-CoV-2), was declared a global pandemic in March 2020.(1) A high body mass index (BMI) has previously been associated in a linear and non-linear fashion with an increased risk of multiple health outcomes such as metabolic and cardiovascular conditions, cancer, viral infections, and mortality.(2–5) A better understanding of the relation between BMI and the progression of COVID-19 is essential for clinical management of patients and implementation of preventive strategies.

A review and meta-analysis of 75 studies indicated obesity (BMI ≥30kg/m$^2$) as a risk factor for severe COVID-19 and related mortality.(6) Additionally, two studies with data from a subsample of the UK Biobank and a New York hospital found that BMI was associated in a dose-response manner with an increased risk of testing positive for SARS-CoV-2 and in a J-shaped fashion with the risk of intubation or death, respectively.(7,8) These studies have provided relevant insights into this association. However, they have certain limitations that include being restricted to tested or hospitalised populations (increasing the risk of collider bias), having a small sample size, limitedly accounting for potential confounding, or dichotomizing BMI (with/without obesity).(9) Some of these limitations difficult the generalization of the studies' conclusions to populations with milder forms of disease or the general population. A study conducted with comprehensive patient-level data containing detailed individuals' BMI information and capturing incident COVID-19 cases from a large and representative population where outcomes are recorded in diverse healthcare settings, could add valuable information to complement the previous evidence in the understanding of the BMI-COVID-19 association.

Catalonia was heavily hit by the first phase (March through May) of the COVID-19 pandemic.(10) This region has a universal taxpayer-funded primary care-based health system in which general practitioners have been the first point of contact for care throughout the pandemic. Electronic health records (EHRs) from primary care encompassing demographic, historical lifestyle information and disease diagnoses linked to SARS-CoV-2 Reverse Transcription Polymerase Chain Reaction (RT-PCR) test results, hospital records, and regional mortality data offer a unique opportunity to study the role of BMI in the course of COVID-19. We aimed to investigate the associations between BMI and risks of COVID-19 diagnosis, hospitalisation with COVID-19, and death after a COVID-19 diagnosis or hospitalisation (*subsequent death*), accounting for potential effect modification by age and sex, using EHR data from Catalonia.

## Methods

### Study design, setting and data sources

We conducted a cohort study from the 1st March to the 6th May 2020. We used prospectively collected primary care records from the Information System for Research in Primary Care (SIDIAP; www.sidiap.org) in Catalonia, Spain. SIDIAP includes data from the *Institut Català de la Salut* (ICS, Catalan Health Institute), the largest public primary healthcare provider of Catalonia (covering 5.8 million people, 80% of the population of Catalonia) since 2006 and is representative of the Catalan population in terms of age, sex, and geographic distribution.(11) SIDIAP includes high-quality data on anthropometric measurements, disease diagnoses, laboratory tests, demographic and lifestyle information. SIDIAP has been linked to COVID-19 RT-PCR test results, hospital records, and regional mortality data, and mapped to the Observational Medical Outcomes Partnership (OMOP) Common Data Model (CDM).(12) The latter allowed to structure the data in a standardised format, and apply analytical tools developed by the open-science Observational Health Data Sciences and Informatics (OHDSI) network.(13)

### Multistate framework

We addressed our objectives using a multi-state framework. Multi-state models allow for a description of the progression from a time origin until the occurrence of several events, extending on competing risk models by also describing transitions to intermediate events.(14) In the context of COVID-19, outpatient diagnoses and hospitalisations with the disease can be considered as intermediate events between not being (identified as) infected on one end to death on the other. We structured our multi-state model in four states: *general population*, *diagnosed* (with COVID-19), *hospitalised* (with COVID-19), and *death* (Figure 1). The following transitions were possible: *general population* to either *diagnosed*, *hospitalised* or *death*; *diagnosed* to either *hospitalised* or *death*; *hospitalised* to *death*. This approach is valuable in the context of the first wave of COVID-19 because it provides a more detailed overview of the interaction between individuals and the health system, respective of their BMI. This framework allows disentangling the association between BMI and risk of hospitalisation with COVID-19 differentiating direct hospitalisations (among the community) from indirect ones (among people already diagnosed with COVID-19 in primary care). Similarly, this approach distinguishes the risk of death related to BMI among individuals who interacted exclusively with primary care (only had an outpatient diagnosis) and those who interacted with secondary care (were hospitalised) before dying. Furthermore, this approach can reduce the risk of collider bias that can be induced by just assessing one transition of interest.(9)

### Participants

We identified all adults (aged ≥18 years) registered in the SIDIAP as of the 1st March 2020 with a BMI recorded at an age ≥18 years. We excluded individuals with more than one year of prior history available (to have sufficient time to capture participants' characteristics before study entry) and with a previous clinical diagnosis or positive test result for COVID-19. We also excluded those who were hospitalised or living in a nursing home on the 1st March 2020, because the transmission dynamics and frequency of testing/diagnosing of these sites differed from the community population, which was the focus of this study.(15,16) Finally, individuals without information on smoking and socioeconomic status were also excluded. The flow chart of inclusion and exclusion criteria for this study is presented in Figure S1 of the Supplementary Material.(17) The descriptive characteristics of the individuals excluded due to living in nursing homes is available in Table S1.(17) Individuals' follow-up period began on the 1st of March 2020 (index date) and ended for any given transition due to exit from the database (which refers to individuals moving out of the catchment area of SIDIAP), the occurrence of the event of interest or a competing event, or the end of the study period.

### Variables

The exposure of interest was BMI as a continuous variable (kg/m$^2$). BMI was calculated using the weight and height of patients assessed in a standardized manner by general practitioners or nurses.(18) The exposure was assigned as the closest valid BMI (≥15kg/m$^2$ and ≤60kg/m$^2$) to the index date recorded between January 1st 2006 and February 29th 2020.

The characteristics of interest were sex, age, smoking status, socioeconomic status, and comorbidities. We extracted participants' sex (female, male), age (in years) at index date and smoking status (never, former or current smoker). We assessed socioeconomic status using the

“Mortalidad en áreas pequeñas Españolas y Desigualdades Socioeconómicas y Ambientales” (MEDEA) deprivation index, which is calculated at the census tract level in urban areas of Catalonia.(19) This measure is categorized into quintiles for anonymization purposes, the first quintile represents the least deprived group of the population and the fifth the most deprived one. It also includes a rural category since the MEDEA index is not available for participants living in those areas. We identified the following comorbidities using the individual’s medical history: autoimmune condition, chronic kidney disease, chronic obstructive pulmonary disease (COPD), heart disease, hyperlipidemia, hypertension, malignant neoplasm (excluding non-melanoma skin cancer) and type 2 diabetes. We selected these conditions based on their relevance to the obesity and COVID-19 research fields and their availability in the OMOP-CDM mapped version of the SIDIAP database and were defined as in a previous study conducted using SIDIAP data.(20–22) The definitions are available in a web application (“Index Event Breakdown” tab) at https://livedataoxford.shinyapps.io/MultiStateCovidCohorts/.

The outcomes of interest were an outpatient (primary care) clinical diagnosis of COVID-19, a hospitalisation with COVID-19, and death. We defined outpatient COVID-19 diagnoses based on a recorded clinical code for COVID-19 disease (International Classification of Diseases, Tenth Revision, Clinical Modification [ICD-10-CM] codes B34.2 “Coronavirus infection, unspecified” and B97.29 “Other coronavirus as the cause of diseases classified elsewhere”). We did not require a positive RT-PCR test result in the definition of clinical diagnoses of COVID-19 due to testing restrictions during the first months of the pandemic.(23) At the time of the study, RT-PCR tests were mostly conducted in patients with severe disease who were, or about to be, hospitalised; progressively with time, specific at-risk populations were also prioritised. Given that the focus of this study was to capture individuals with COVID-19 in the general population, we considered the clinical diagnosis as reported in the SIDIAP. We defined hospitalisation with COVID-19 as a hospital admission (hospital stay of at least one night) where the individual had a positive RT-PCR test result or a clinical diagnosis of COVID-19 over the 21 days before their admission up to the end of their hospital stay. We defined mortality using region-wide mortality data, and so included both deaths during hospitalisations and in the community.

### Statistical analyses

We reported the participants' baseline characteristics by World Health Organization (WHO) categories of BMI (underweight [BMI <18.5 kg/m$^2$], normal weight [BMI ≥18.5 and <25 kg/m$^2$], overweight [BMI ≥25 and <30kg/m$^2$] and obesity [BMI ≥30kg/m$^2$]).

We compared the baseline characteristics of the included individuals to those of the excluded due to unavailability of BMI, smoking status and/or the MEDEA deprivation index information using standardized mean differences (SMDs). We considered an |SMD| >0.1 indicate meaningful differences in the distribution of a given characteristic between the two groups.(24)

We described the participants' time at risk at each state and the absolute number of outcomes observed for each transition, by WHO categories of BMI. We assessed the relationship between BMI and the risk of transitioning to a subsequent state in the multistate model by estimating cause-specific hazard ratios (HRs) and 95% confidence intervals (CIs) using Cox proportional hazard regressions. We estimated three types of models: 1) with BMI as the sole explanatory variable (unadjusted models); 2) adjusted for age and sex; 3) adjusted for age, sex, smoking status and the MEDEA deprivation index (fully adjusted models). We used a directed acyclic graph to guide decisions on the control for confounding (Figure S2 of the Supplementary Material).(17,25) We considered non-linearity in BMI and transitions by fitting models with BMI as a linear term, with a polynomial of degree 2 (i.e. quadratic), and with restricted cubic splines (with 3, 4, or 5 knots).(26) We calculated the Bayesian Information Criterion (BIC) and we favoured the model with the lowest BIC values. We compared the model where BMI was fitted with a non-linear term against a linear model using a likelihood ratio test. We fitted age in the adjusted models using the same strategy as for BMI. We checked the proportional hazard assumptions for the variables included in the models by visual inspection of log-log survival curves. We did not model the transition from the *general population* to *death* because we were interested in deaths related to COVID-19 (*subsequent deaths*) which we captured by having gone through the *diagnosed* or *hospitalised* states (Figure 1). However, we considered *death* among the *general population* as a competing risk by censoring people at their death.

We assessed effect modification by introducing interaction terms (one at a time) between BMI and age and sex. We stratified the models in three categories of age (18-59, 60-79, and ≥80 years) and sex. As secondary analyses, we re-estimated the models fitting BMI in WHO categories and we assessed the effect of obesity-related comorbidities (hypertension, type 2 diabetes and hyperlipidemia) in the studied associations by introducing interaction terms (one at a time) between BMI and each comorbidity.

For the main analyses, we conducted a complete case analysis (we only included individuals with complete information on BMI and the covariates of interest). To explore the possibility of selection bias due to excluding those with missing data, in a sensitivity analysis we re-estimated the main models after multiple imputations (using predictive mean matching, with 5 imputations drawn) of missing data on BMI, smoking status, and/or the MEDEA deprivation index. The variables used for the multiple imputations were BMI, sex, age, smoking status, the MEDEA deprivation index, time of follow-up for each transition and outcomes of interest (given that we are using a time to event analysis) the Charlson comorbidity index, and a wide range of health conditions.(27,28) In a second sensitivity analysis, we considered the impact of exposure misclassification. We replicated the main analyses firstly including only BMI values recorded in the previous five years (March 1st 2015 to February 29th 2020) and secondly including only BMI values recorded in the previous two years (March 1st 2018 to February 29th 2020).

We used R version 3.6 for data analysis and visualization. The R packages used for the analyses included numerous tidyverse packages, mstate, survival and rms.(29–32) The analytic code we used is available at https://github.com/SIDIAP/MultiStateBmiCovid-19.

This study was approved by the Clinical Research Ethics Committee of the IDIAPJGol (project code: 20/070-PCV).

## Results

There were 4 765 757 adults from the SIDIAP population registered in the database on the 1st March 2020 who were eligible to enter the study. We excluded 104 022 individuals due to having less than a year of prior clinical history; 306 to having a prior COVID-19 clinical diagnosis or positive test; 41 588 to being hospitalised or living in a nursing home on March 1st; 1 357 553 to the unavailability of a BMI measurement; and 737 362 to missing data on smoking status and/or MEDEA deprivation index (Figure S1 of the Supplementary Material).(17) We included 2 524 926 participants, of which 45 382 were living with underweight (2%), 905 898 with normal weight (36%), 952 479 (38%) with overweight, and 621 167 (24%) with obesity (Table 1). The participants' median BMI (interquartile [IQR] range) was 26 (24-30) kg/m$^2$ and age was 52 (39-67) years. People living with underweight or normal weight were younger and more frequently female, current smokers, living in the least deprived areas of Catalonia and presenting with fewer comorbidities than people living with overweight or obesity (Table 1).

All the analysed baseline characteristics of the included individuals were meaningfully different (SMDs >0.1) from those of the excluded individuals due to missing information on BMI, smoking status and/or the MEDEA deprivation index (Table S2 of the Supplementary Material).(17) Especially, the included participants were older (median age: 52 vs 44 years), more commonly female (55% vs 47%) and more frequently presenting with comorbidities (e.g., hypertension prevalence: 20% vs 8%).

After a median follow-up of 67 days of the general population, 57 443 (2.28%) were diagnosed with COVID-19 (median [IQR] BMI: 27 [24-30]kg/m$^2$) and 5191 (0.21%) were hospitalised without a prior COVID-19 outpatient diagnosis (29 [26-32]kg/m$^2$) (Tables 2 and S3). Among the people diagnosed with COVID-19 in outpatient settings, 5671 (10.62%) went on to be hospitalised (28 [26-32]kg/m$^2$) and 1166 (2.43%) died (27 [24-30]kg/m$^2$) (median follow-up: 35 days). Finally, of the people that were hospitalised with COVID-19, 1301 (19.22%) died (29 [26-32]kg/m$^2$) (median follow-up: 37 days). The time at risk and absolute event rates of the participants by WHO categories of BMI are shown in Table 2 and the descriptive characteristics of people transitioning to each state are available in Table S3 of the Supplementary Material.(17)

BMI was non-linearly associated with the risk of COVID-19 diagnosis, hospitalisation with COVID-19, and *subsequent death* for all studied transitions in the fully adjusted models (all p for non-linearity ≤0.001) (Figure 2). Results for the crude and adjusted for age and sex models are shown in Figure S3 and Table S4 of the Supplementary Material; the latter were similarly shaped to the fully adjusted models.(17) There was a modest positive association between BMI and the risk of COVID-19 *diagnosis* (Figure 2). Relative to a BMI of 22kg/m$^2$, the estimated HRs were 0.81 (0.79-0.84) for someone with a BMI of 16kg/m$^2$; 1.10 (1.09-1.11) for a BMI of 25kg/m$^2$; 1.22 (1.19-1.24) for a BMI of 31kg/m$^2$, and 1.28 (1.25-1.32) for a BMI of 40kg/m$^2$ (Table 3).

BMI was strongly associated with an increased risk of *hospitalisation* with COVID-19, either with or without a prior outpatient diagnosis (Figure 2). Hazard ratios for hospitalisation without and with a prior diagnosis respectively, relative to a BMI of 22kg/m$^2$, were 0.58 (0.53-0.64) and 0.51 (0.46-0.57) for a BMI of 16kg/m$^2$; 1.27 (1.22-1.31) and 1.37 (1.31-1.43) for one of 25kg/m$^2$; 1.88 (1.75-2.03) and 2.01 (1.86-2.18) for one of 31kg/m$^2$; 2.85 (2.58-3.13) and 2.66 (2.43-2.91) for one of 40kg/m$^2$ (Table 3).

The association between BMI and risk of *death* either after an outpatient diagnosis or a hospitalisation with COVID-19 was J-shaped (Figure 2). Relative to a BMI of 22kg/m$^2$, a BMI of 16kg/m$^2$ was associated with HRs of 1.28 (1.07-1.52) and 1.20 (1.02-1.42) for death after an outpatient diagnosis or a hospitalisation with COVID-19, respectively (Table 3). High BMIs became positively associated with death only at BMIs ≥37kg/m$^2$ among those previously hospitalised (HR [95% CI]: 1.26 [1.06-1.51]) and 40kg/m$^2$ among those diagnosed in outpatient settings (1.27 [1.03-1.56]).

There was evidence of effect modification by age and sex for four out of five studied transitions (p for interaction <0.001) (Figure 3). The risk of COVID-19 outcomes related to increased BMI was higher for those aged ≤59 years, compared to those in older age groups (Figure 3, Table S5 of the Supplementary Material).(17) Also, the risk of COVID-19 diagnosis for BMIs ≥40kg/m$^2$ was higher for the oldest age group (HR, [95% CI]: 1.52 [1.33-1.74], relative to a BMI of 22kg/m$^2$) compared to those aged between 60 and 79 (1.10 [1.03-1.18]) or ≤59 years (1.32 [1.28-1.37]) (Figure 3, Table S5 A of the Supplementary Material).(17) BMI was not associated with mortality after an outpatient diagnosis for those in the oldest age group, but there was a pronounced U-shaped association for those aged ≤59 years and a J-shaped association for those aged between 60 and 79 years (Figure 3, Table S5 A of the Supplementary Material).(17) Associations were similarly shaped for females and males, although males were at a slightly higher risk of being diagnosed or hospitalised with COVID-19 compared to females (Figure 3, Table S5 B of the Supplementary Material).(17) The risk of death after hospitalisation with COVID-19 was stronger for females with BMIs ≥43kg/m$^2$ (2.23 [1.66-3.00] relative to a BMI of 22kg/m$^2$) compared to males (1.30 [0.92-1.85]) (Figure 3, Table S5 B of the Supplementary Material).(17)

The assumption of proportionality was violated for age in the first transition. To account for this, we stratified the main model by calendar month. The risk of COVID-19 diagnosis related to increased BMI was slightly higher for those diagnosed in March compared to April (Figure S4, Table S6 of the Supplementary Material).(17)

As a first secondary analysis, we re-estimated the main models with BMI in WHO categories (Figure S5 of the Supplementary Material).(17) Relative to normal weight, overweight and obesity were associated with a higher risk of being diagnosed and hospitalised with COVID-19; no statistically significant associations were observed for the underweight category. No association between categorized BMI and risk of *subsequent death* was observed. As a second secondary analysis, we assessed the effect of comorbidities in the association between BMI and COVID-19 outcomes. The positive association between BMI and risks of COVID-19 diagnosis and COVID-19 hospitalisation

(with and without a prior outpatient diagnosis) was higher for individuals without hypertension compared to those with hypertension (p for interaction <0.01) (Figure S6 of the Supplementary Material).(17) A similar pattern was observed for people without type 2 diabetes for whom the association between BMI and risk of COVID-19 hospitalisation (with and without a prior outpatient diagnosis) was higher than for those with type 2 diabetes (p for interaction <0.01) (Figure S7 of the Supplementary Material).(17) Finally, individuals without hyperlipidemia had a modestly higher risk of COVID-19 diagnosis compared to those with hyperlipidemia (Figure S8 of the Supplementary Material).(17)

Our findings were robust to two sensitivity analyses. The shape of the studied associations and the estimated effect sizes of our main analyses were similar to those of the analyses in which we did multiple imputations on missing data for BMI and the model's covariates and in which we excluded BMI measurements older than five or two years (Figures 2, S9-S11 and Tables 3, S7-S9), with 2 041 652 and 1 405 484 individuals included in each analysis, respectively.(17) Nevertheless, the association between BMI and death after a COVID-19 diagnosis or a COVID-19 hospitalisation was attenuated in the analyses of the multiple imputations.

## Discussion

In this large cohort study that included 2 524 926 participants from the general population in Catalonia, we found a monotonic association between BMI and COVID-19 diagnosis and hospitalisation risks and a J-shaped one with mortality. The associations between BMI and COVID-19 outcomes were stronger for those aged ≤59 years and similarly shaped among females and males, with specific exceptions.

The strengths of this study include being a large longitudinal study that investigates the association between BMI and the course of the COVID-19 disease containing individual detailed BMI information and incident COVID-19 outcomes recorded in diverse healthcare settings from a large and representative population. Also, the possibility to investigate COVID-19 trajectories in a single and sufficiently powered dataset, including systematic investigation of non-linearity and effect modification, is a major strength. Further, our results were robust when we explored the violation of the models' assumptions, the possibility of selection bias and exposure misclassification.

This study also has weaknesses. The exposure was captured using a 14-year window, which for certain individuals relied on the assumption that BMI measurements were constant for a long period. However, we observed that the median of time elapsed since the BMI measurement was 1.7 years (interquartile range: 0.6 to 4.0) for the included participants. Moreover, in the sensitivity analyses where we used BMI measurements that were no older than five or two years the obtained results were very similar to those of the main analysis. We defined COVID-19 cases as individuals who had a clinical diagnosis of the disease. Although this could have resulted in false positives, we decided not to require a confirmation of an RT-PCR positive test because testing was mainly restricted to severe cases of COVID-19 and specific at-risk populations during the first wave of the pandemic. This decision resulted in including only COVID-19 diagnoses of individuals who interacted with the health system, missing asymptomatic patients or individuals who did not seek medical care. However, Catalonia has a tax-funded almost universal healthcare system. Further, the results of this study are not generalizable to people living in nursing homes since we decided to exclude this sub-group of the population. We did not have the cause of death (only death after being diagnosed/hospitalised with COVID-19) which prevented us from attributing deaths to the disease. However, *subsequent deaths* were more frequent and happened more quickly than the deaths among the general population. The cumulative incidence of death was 0.2% in the general population, compared to 2.4% and 19.2% in those diagnosed and hospitalised with COVID-19, respectively (Table 2). The median time to death after a COVID-19 diagnosis or hospitalisation was much shorter (35 and 37 days, respectively) than for those in the general population (67 days) (Figure S12), which suggests *subsequent deaths* were COVID-19 related.(17) Additionally, we will have missed individuals who died with COVID-19 but who were not identified as having been diagnosed or hospitalised with the disease. The likelihood of this outcome misclassification was probably reduced with the exclusion of nursing homes' residents. We did not have data on hospital visits that did not lead to an overnight stay nor admission to intensive services units; this data can be useful to further study the progression of COVID-19 in detail. We did not have information on individual socioeconomic status nor the type of occupation of the participants; we tried to minimize this limitation by including the MEDEA deprivation index. Finally, the use of routinely collected data for research can raise concerns about data quality; however, BMI and COVID-19 data from the SIDIAP have successfully been repurposed for research.(22,33,34)

The mechanisms by which higher BMI can increase COVID-19 severity include physical mechanisms (e.g., altered ventilation due to reduced diaphragm excursion), chronic inflammation and impaired immune function.(6) Higher BMI is also a risk factor for several medical conditions that could mediate the association between adiposity and the risk of COVID-19 severity such as type 2 diabetes or hypertension (which were also common in this study among patients with obesity).(6,21) Our findings support the latter hypothesis: the positive association between BMI and the risk of being hospitalised with COVID-19 was attenuated among people with hypertension or type 2 diabetes (compared to those without). This suggests that shared biological mechanisms between obesity, hypertension and type 2 diabetes might partially explain the higher susceptibility to COVID-19 hospitalisation among individuals living with these conditions. Other proposed explanations include delayed seek for medical care among individuals with obesity due to fear of stigmatization (e.g., 26%

and 39% of those diagnosed and hospitalised without an outpatient diagnosis of COVID-19, respectively, had obesity) and the difficulty of care in hospital settings for supportive therapies.(35,36)

Obesity has been associated with the risk of SARS-CoV-2 infection and COVID-19 diagnosis.(6) Our dose-response analysis revealed that the risk of COVID-19 diagnosis increased linearly with higher BMI values, which is in line with a study of UK Biobank participants.(8) Our findings are also aligned with a Mendelian randomization analysis which reported that genetically increased BMI was causally associated with COVID-19 positivity.(37) These results highlight the importance of avoiding extremely high BMI cut-offs to determine vulnerable groups to the COVID-19 disease (e.g., the NHS only considers BMIs >40kg/m$^2$ as risk groups).(20)

Our findings revealed a much stronger association between BMI and COVID-19 diagnosis among those aged ≥80 years and a modestly higher risk among males. While our findings are congruent with another study of the UK Biobank regarding sex differences in risk, no effect modification by age group (younger vs older than 70 years) was reported there.(38) The underlying age distribution of those participants could explain this discrepancy; unfortunately, this information was unavailable.

Our findings of a strong positive association between BMI and risk of COVID-19 hospitalisation are in line with a large meta-analysis and a population-based study conducted in another Spanish region (Navarra).(6,39) Our results also suggest the necessity to lower BMI cut-offs to establish risk groups for disease severity.

The risk of hospitalisation with COVID-19 was systematically higher for those aged ≤59 years which is congruent with two hospital-based studies from the US. One reported a negative correlation between BMI and age among COVID-19 patients in six hospitals and another a positive association only among patients aged <60 years compared to older adults.(40,41) Further, these results are congruent with the above-mentioned study from Navarra, where it was reported that the association between severe obesity and risk of hospitalisation was much higher for those aged between 25 and 49 years.(39)

Two meta-analyses reported that obesity is associated with a higher risk of COVID-19 mortality.(6,42) However, non-linear associations cannot be ignored in BMI-related research, especially concerning mortality.(4,5) Large observational studies from the US, the UK and Spain using multiple categories of BMI only found an association between morbid obesity (BMIs >35 or 40kg/m$^2$) and COVID-19 mortality.(39,43–46) Our results for high BMIs are consistent with the latter studies and revealed BMI was associated in a J-shaped fashion with the risk of *subsequent death*. Only BMIs above 37kg/m$^2$ and 40kg/m$^2$ were linked with a higher risk of death after a COVID-19 hospitalisation and after a COVID-19 outpatient diagnosis, respectively. The J-shaped association between BMI and risk of COVID-19 related death has also been reported in a study conducted in a New York hospital and England using a large primary care database.(7,47) Interestingly, we observed a lower risk of death for individuals living with overweight among people diagnosed with COVID-19 in outpatient settings. These results are congruent with the English study which also reported HRs below 1 for the risk of confirmed or suspected deaths due to COVID-19 in BMIs in the overweight range.(47) Our results provide important insights on the higher risk of *subsequent death* for low BMIs (≤19kg/m$^2$); while other studies also found this trend, these were not significant, likely due to their smaller sample sizes.(7,45,46)

We also found that mortality risk related to an increased BMI was higher among individuals aged ≤69 years compared to older adults. Four previous studies are much in line with our findings, while a meta-analysis reported the opposite.(7,38,42,45,46) The risk of death after a hospitalisation with COVID-19 associated with BMI was higher among females which is congruent with a UK Biobank study.(38) However, a study performed in a New York hospital found a higher risk among males and others found opposite or null differences by sex.(7,42,45,46)

We provided a comprehensive analysis of the association between BMI and the course of COVID-19 during the first wave of the pandemic in Catalonia. Our analyses revealed that BMI is positively associated with being diagnosed and hospitalised with COVID-19, and in a J-shaped fashion with the risk of death following a COVID-19 diagnosis or hospitalisation; the associations were particularly pronounced among younger patients. These findings highlight the necessity to consider individuals with both overweight and obesity as vulnerable groups to COVID-19 and its severity. Defining this high-risk group is especially important for the prioritisation of individuals in preventive strategies such as vaccination campaigns. More broadly, our results reinforce the need for public health strategies focusing on the reduction of overweight and obesity which can help prevent COVID-19 outcomes but also other well-established obesity-related diseases such as cardiometabolic conditions and certain cancer types.

### Acknowledgements

We would like to acknowledge the patients who suffered from or died of this devastating disease, and their families and carers. We would also like to thank the healthcare professionals involved in the management of COVID-19 during these challenging times, from primary care to intensive care units in the Catalan healthcare system.

### Data availability

In accordance with current European and national law, the data used in this study is only available for the researchers participating in this study. Thus, we are not allowed to distribute or make publicly available the data to other parties. However, researchers from public institutions can request data from SIDIAP if they comply with certain requirements. Further information is available online (https://www.sidiap.org/index.php/menu-solicitudesen/application-proccedure) or by contacting Anna Moleras (amoleras@idiapjgol.org).

### Code availability

We used R version 3.6 for data analysis and visualization. The R packages used for the analyses included numerous tidyverse packages, mstate, survival and rms. The analytic code we used is available at https://github.com/SIDIAP/MultiStateBmiCovid-19.

### Authors' contributions

SFB, MA, TDS mapped source data to the OMOP-CDM. MR, AP, EB and TDS led the data analysis. MR and HF performed a literature review. All authors were involved in the study conception and design, interpretation of the results, and the preparation of the manuscript.

### Ethics approval

This study was approved by the Clinical Research Ethics Committee of the IDIAPJGol (project code: 20/070-PCV).

### Disclaimer

Where authors are identified as personnel of the International Agency for Research on Cancer / World Health Organization, the authors alone are responsible for the views expressed in this article and they do not necessarily represent the decisions, policy or views of the International Agency for Research on Cancer / World Health Organization.

## Abbreviations

BIC: Bayesian Information Criterion; BMI: body mass index; CDM: Common Data Model; CI: confidence interval; COPD: chronic obstructive pulmonary disease; COVID-19: coronavirus disease 2019; EHR: Electronic Health Record; HR: hazard ratio; ICD-10-CM: International Classification of Diseases, Tenth Revision, Clinical Modification; IQR: Interquartile Range; MEDEA: Mortalidad en áreas pequeñas Españolas y Desigualdades Socioeconómicas y Ambientales; OMOP: Observational Medical Outcomes Partnership; OHDSI: Observational Health Data Sciences and Informatics; RT-PCR: Reverse Transcription Polymerase Chain Reaction; SARS-CoV-2: Severe Acute Respiratory Syndrome Coronavirus 2; SIDIAP: Information System for Research in Primary Care; SMD: Standardized Mean Difference; WHO: World Health Organization.

## Legends for Figures and Tables

**Figure 1.** Overview of the multi-state model which provided the framework for this study

Notes: The transition from general population to death was used for censoring, but was not a transition of interest in modelling (grey dashed lines). The percentages depicted between transitions correspond to the cumulative incidence at 65 (for those in the general population), and 45 days (for those diagnosed and hospitalised with COVID-19).

Abbreviations: BMI: Body Mass Index; COVID-19: Coronavirus Disease 2019

**Table 1.** Descriptive statistics of the study population by body mass index categories

Notes: BMI categories: underweight (BMI <18.5 kg/$m^2$), normal weight (BMI ≥18.5 and <25 kg/$m^2$), overweight (BMI ≥25 and <30 kg/$m^2$) and obesity (BMI ≥30 kg/$m^2$). Malignant neoplasm does not include non-melanoma skin cancer.

Abbreviations: BMI: Body Mass Index; COPD: Chronic Obstructive Pulmonary Disease; IQR: Interquartile range; MEDEA: "Mortalidad en áreas pequeñas Españolas y Desigualdades Socioeconómicas y Ambientales".

**Table 2.** Time at risk, absolute event rates, and cumulative incidence over time by body mass index categories

Notes: BMI categories: underweight (BMI <18.5 kg/$m^2$), normal weight (BMI ≥18.5 and <25 kg/$m^2$), overweight (BMI ≥25 and <30 kg/$m^2$) and obesity (BMI ≥30 kg/$m^2$). 22.2% (12,730/57,443) of individuals diagnosed with COVID-19 also had a positive RT-PCR test result.

Abbreviations: BMI: Body Mass Index; COVID-19: Coronavirus Disease 2019; IQR: Interquartile range.

**Figure 2.** Association between body mass index and the risk of COVID-19 outcomes, allowing for non-linear effects, with 95% Cis

Notes: Models are adjusted for age, sex, smoking status and the MEDEA deprivation index. P-values for non-linearity were obtained by comparing the model where BMI was fitted with a non-linear term against a linear model using a likelihood ratio test.

Abbreviations: BMI: Body mass index; CI: Confidence interval; COVID-19: Coronavirus Disease 2019.

**Table 3.** Hazards ratios of COVID-19 outcomes related to body mass index, with 95% Cis

Notes: Models are adjusted for age, sex, smoking status and the MEDEA deprivation index.

Abbreviations: BMI: Body mass index; CI: Confidence interval; COVID-19: Coronavirus Disease 2019.

**Figure 3.** Effect modification by age and sex in the association between body mass index and the risk of COVID-19 outcomes, allowing for non-linear effects, with 95% CIs

Notes: Models are adjusted for age, sex, smoking status and the MEDEA deprivation index. P-values for interaction were obtained by comparing the fully adjusted model which included an interaction term (left side of the figure with age, right side with sex) against the fully adjusted model using a likelihood ratio test.

Abbreviations: BMI: Body mass index; CI: Confidence interval; COVID-19: Coronavirus Disease 2019.

## Tables

**Table 3.** Hazards ratios of COVID-19 outcomes related to body mass index, with 95% CIs

| | | BMI categories | | | |
|---|---|---|---|---|---|
| | **Overall** | **Underweight** | **Normal weight** | **Overweight** | **Obesity** |
| **N** | 2 524 926 | 45 382 | 905 898 | 952 479 | 621 167 |
| **BMI, median (IQR)** | 26 (23.5-29.9) | 17.8 (17.2-18.2) | 22.7 (21.2, 23.9) | 27 (26.1-28.5) | 33 (31.2-35.8) |
| **Time elapsed since BMI measurement, median (IQR)** | 1.7 (0.6-4.0) | 2.5 (1.0-5.1) | 2.3 (0.9-5.0) | 1.6 (0.5-3.8) | 1.0 (0.3-2.7) |
| **Age, median (IQR)** | 52 (39-67) | 35 (26-48) | 45 (34-60) | 56 (43-70) | 58 (45-70) |
| **Age, n (%)** | | | | | |
| 18 to 39 | 633 408 (25.1) | 27 552 (60.7) | 330 538 (36.5) | 175 824 (18.5) | 99 494 (16.0) |
| 40 to 59 | 958 492 (38.0) | 11 362 (25.0) | 348 439 (38.5) | 363 878 (38.2) | 234 813 (37.8) |
| 60 to 69 | 405 640 (16.1) | 2605 (5.7) | 100 354 (11.1) | 173 258 (18.2) | 129 423 (20.8) |
| 70 to 79 | 325 948 (12.9) | 1670 (3.7) | 70 900 (7.8) | 148 561 (15.6) | 104 817 (16.9) |
| 80 or older | 201 438 (8.0) | 2193 (4.8) | 55 667 (6.1) | 90 958 (9.5) | 52 620 (8.5) |
| **Female sex, n (%)** | 1 386 678 (54.9) | 35 139 (77.4) | 549 089 (60.6) | 454 195 (47.7) | 348 255 (56.1) |
| **Smoking status, n (%)** | | | | | |

| | | | | | |
|---|---|---|---|---|---|
| Never smoker | 1 343 985 (53.2) | 23 568 (51.9) | 489 677 (54.1) | 503 401 (52.9) | 327 339 (52.7) |
| Former smoker | 663 383 (26.3) | 6067 (13.4) | 186 830 (20.6) | 274 962 (28.9) | 195 524 (31.5) |
| Current smoker | 517 558 (20.5) | 15 747 (34.7) | 229 391 (25.3) | 174 116 (18.3) | 98 304 (15.8) |
| **MEDEA deprivation index, n (%)** | | | | | |
| Quintile 1 (least deprived) | 394 503 (15.6) | 8706 (19.2) | 168 140 (18.6) | 143 760 (15.1) | 73 897 (11.9) |
| Quintile 2 | 399 883 (15.8) | 7325 (16.1) | 149 848 (16.5) | 151 283 (15.9) | 91 427 (14.7) |
| Quintile 3 | 405 747 (16.1) | 7019 (15.5) | 141 866 (15.7) | 155 003 (16.3) | 101 859 (16.4) |
| Quintile 4 | 410 440 (16.3) | 6735 (14.8) | 135 637 (15.0) | 156 861 (16.5) | 111 207 (17.9) |
| Quintile 5 (most deprived) | 410 231 (16.2) | 7134 (15.7) | 130 020 (14.4) | 153 535 (16.1) | 119 542 (19.2) |
| Rural | 504 122 (20.0) | 8463 (18.6) | 180 387 (19.9) | 192 037 (20.2) | 123 235 (19.8) |
| **Comorbidities, n (%)** | | | | | |
| Autoimmune condition | 170 240 (6.7) | 2575 (5.7) | 52 165 (5.8) | 63 801 (6.7) | 51 699 (8.3) |
| Chronic kidney disease | 141 921 (5.6) | 956 (2.1) | 30 583 (3.4) | 61 692 (6.5) | 48 690 (7.8) |
| COPD | 86 723 (3.4) | 1340 (3.0) | 21 163 (2.3) | 35 105 (3.7) | 29 115 (4.7) |
| Heart disease | 363 012 (14.4) | 2975 (6.6) | 85 868 (9.5) | 153 188 (16.1) | 120 981 (19.5) |
| Hyperlipidemia | 357 572 (14.2) | 2099 (4.6) | 86 455 (9.5) | 157 070 (16.5) | 111 948 (18.0) |

| | | | | | |
|---|---|---|---|---|---|
| Hypertension | 514 533 (20.4) | 2165 (4.8) | 96 289 (10.6) | 220 109 (23.1) | 195 970 (31.5) |
| Malignant neoplasm | 197 171 (7.8) | 2109 (4.6) | 56 734 (6.3) | 84 503 (8.9) | 53 825 (8.7) |
| Type 2 diabetes | 236 253 (9.4) | 656 (1.4) | 34 065 (3.8) | 94 963 (10.0) | 106 569 (17.2) |
| **Cause of end of follow-up, n (%)** | | | | | |
| End of study | 2 515 630 (99.6) | 45 131 (99.4) | 902 920 (99.7) | 948 894 (99.6) | 618 685 (99.6) |
| Transferred-out of the SIDIAP | 7743 (0.3) | 207 (0.5) | 2347 (0.3) | 3035 (0.3) | 2154 (0.3) |
| Death | 1553 (0.1) | 44 (0.1) | 631 (0.1) | 550 (0.1) | 328 (0.1) |

| | From general population | | | | | From diagnosed with COVID-19 | | | | From hospitalised with COVID-19 | | |
|---|---|---|---|---|---|---|---|---|---|---|---|---|
| | | | To diagnosis with COVID-19 | To hospitalised with COVID-19 | To death | | | To hospitalised with COVID-19 | To death | | | To death |
| BMI Categories | n | Follow-up in days, Median (min, IQR, max) | Events (cumulative incidence at 67 days) | Events (cumulative incidence at 67 days) | Events (cumulative incidence at 67 days | n | Follow-up in days, Median (min, IQR, max) | Events (cumulative incidence at 45 days) | Events (cumulative incidence at 45 days) | n | Follow-up in days, Median (min, IQR, max) | Events (cumulative incidence at 45 days) |
| **Overall** | 2 524 926 | 67 (1, 67 to 67, 67) | 57 443 (2.28%) | 5191 (0.21%) | 5276 (0.21%) | 57 443 | 35 (0, 19 to 44, 66) | 5671 (10.26%) | 1166 (2.43%) | 10 862 | 37 (0, 27 to 43, 65) | 1301 (19.22%) |
| **Underweight** | 45 382 | 67 (2, 67 to 67, 67) | 991 (2.18%) | 34 (0.08%) | 176 (0.39%) | 991 | 36 (0, 21.5 to 44, 58) | 25 (2.62%) | 23 (2.64%) | 59 | 36 (3, 23 to 44.5, 61) | 8 (25.25%) |
| **Normal weight** | 905 898 | 67 (1, 67 to 67, 67) | 19 940 (2.20%) | 905 (0.10%) | 1731 (0.19%) | 19 940 | 37 (0, 21 to 44, 66) | 1068 (5.61%) | 355 (2.62%) | 1973 | 35 (0, 24 to 42, 65) | 261 (22.62%) |
| **Overweight** | 952 479 | 67 (1, 67 to 67, 67) | 21 369 (2.24%) | 2233 (0.23%) | 2024 (0.21%) | 21 369 | 35 (0, 17 to 44, 66) | 2421 (11.76%) | 470 (2.64%) | 4654 | 37 (0, 27 to 43, 65) | 541 (20.72%) |
| **Obesity** | 621 167 | 67 (1, 67 to 67, 67) | 15 143 (2.44%) | 2019 (0.33%) | 1345 (0.22%) | 15 143 | 34 (0, 15 to 43, 66) | 2157 (14.76%) | 318 (2.51%) | 4176 | 37 (0, 28 to 43, 65) | 491 (19.10%) |

| | From General Population | | From diagnosed with COVID-19 | | From hospitalised with COVID-19 |
|---|---|---|---|---|---|
| **BMI values (kg/m$^2$)** | **To diagnosed with COVID-19** | **To hospitalised with COVID-19** | **To hospitalised with COVID-19** | **To death** | **To death** |
| **16** | 0.81 (0.79-0.84) | 0.58 (0.53-0.64) | 0.51 (0.46-0.57) | 1.28 (1.07-1.52) | 1.20 (1.02-1.42) |
| **19** | 0.90 (0.89-0.91) | 0.77 (0.74-0.81) | 0.71 (0.68-0.75) | 1.13 (1.04-1.23) | 1.08 (1.00-1.16) |
| **22** | reference | reference | reference | reference | reference |
| **25** | 1.10 (1.09-1.11) | 1.27 (1.22-1.31) | 1.37 (1.31-1.43) | 0.90 (0.84-0.97) | 0.97 (0.91-1.03) |
| **28** | 1.17 (1.15-1.19) | 1.56 (1.47-1.66) | 1.74 (1.61-1.87) | 0.88 (0.78-0.99) | 0.97 (0.88-1.08) |
| **31** | 1.22 (1.19-1.24) | 1.88 (1.75-2.03) | 2.01 (1.86-2.18) | 0.93 (0.82-1.05) | 1.02 (0.89-1.17) |
| **34** | 1.24 (1.22-1.26) | 2.22 (2.04-2.41) | 2.22 (2.06-2.40) | 1.02 (0.89-1.17) | 1.11 (0.95-1.31) |
| **37** | 1.26 (1.23-1.29) | 2.54 (2.33-2.78) | 2.43 (2.24-2.64) | 1.14 (0.97-1.34) | 1.26 (1.06-1.51) |
| **40** | 1.28 (1.25-1.32) | 2.85 (2.58-3.13) | 2.66 (2.43-2.91) | 1.27 (1.03-1.56) | 1.49 (1.23-1.81) |
| **43** | 1.31 (1.26-1.36) | 3.11 (2.77-3.49) | 2.91 (2.62-3.23) | 1.42 (1.10-1.83) | 1.83 (1.47-2.29) |
| **47** | 1.34 (1.28-1.40) | 3.37 (2.87-3.96) | 3.27 (2.88-3.72) | 1.64 (1.18-2.27) | 2.56 (1.92-3.41) |
| **50** | 1.36 (1.29-1.44) | 3.48 (2.81-4.31) | 3.58 (3.09-4.15) | 1.82 (1.24-2.68) | 3.45 (2.41-4.96) |

Figure 1

Figure 2

Figure 3
